# abc4pwm: affinity based clustering for position weight matrices in applications of DNA sequence analysis

**DOI:** 10.1186/s12859-022-04615-z

**Published:** 2022-03-03

**Authors:** Omer Ali, Amna Farooq, Mingyi Yang, Victor X. Jin, Magnar Bjørås, Junbai Wang

**Affiliations:** 1 Department of Pathology, Oslo University Hospital - Norwegian Radium Hospital, Oslo, Norway; 2 Department of Clinical Molecular Biology, Institute of Clinical Medicine, University of Oslo, Oslo, Norway; 3 Department of Medical Biochemistry, Oslo University Hospital and University of Oslo, Oslo, Norway; 4 Department of Microbiology, Oslo University Hospital and University of Oslo, Oslo, Norway; 5 Department of Molecular Medicine, University of Texas Health San Antonio, San Antonio, TX USA; 6 Department of Clinical Molecular Biology (EpiGen), Akershus University Hospital, Lørenskog, Norway; 7 Department of Clinical and Molecular Medicine, Norwegian University of Science and Technology, Trondheim, Norway

**Keywords:** Transcription factor, Position weight matrices, DNA-binding domain, DNA sequence analysis, Clustering quality assessment, Motif searching

## Abstract

**Background:**

Transcription factor (TF) binding motifs are identified by high throughput sequencing technologies as means to capture Protein-DNA interactions. These motifs are often represented by consensus sequences in form of position weight matrices (PWMs). With ever-increasing pool of TF binding motifs from multiple sources, redundancy issues are difficult to avoid, especially when every source maintains its own database for collection. One solution can be to cluster biologically relevant or similar PWMs, whether coming from experimental detection or in silico predictions. However, there is a lack of efficient tools to cluster PWMs. Assessing quality of PWM clusters is yet another challenge. Therefore, new methods and tools are required to efficiently cluster PWMs and assess quality of clusters.

**Results:**

A new Python package Affinity Based Clustering for Position Weight Matrices (abc4pwm) was developed. It efficiently clustered PWMs from multiple sources with or without using DNA-Binding Domain (DBD) information, generated a representative motif for each cluster, evaluated the clustering quality automatically, and filtered out incorrectly clustered PWMs. Additionally, it was able to update human DBD family database automatically, classified known human TF PWMs to the respective DBD family, and performed TF motif searching and motif discovery by a new ensemble learning approach.

**Conclusion:**

This work demonstrates applications of abc4pwm in the DNA sequence analysis for various high throughput sequencing data using ~ 1770 human TF PWMs. It recovered known TF motifs at gene promoters based on gene expression profiles (RNA-seq) and identified true TF binding targets for motifs predicted from ChIP-seq experiments. Abc4pwm is a useful tool for TF motif searching, clustering, quality assessment and integration in multiple types of sequence data analysis including RNA-seq, ChIP-seq and ATAC-seq.

**Supplementary Information:**

The online version contains supplementary material available at 10.1186/s12859-022-04615-z.

## Background

The complex yet crucial gene regulatory mechanisms to a large extent depend on transcription factors (TFs). TFs read the regulatory signals embedded in the genome and facilitate transmission of these signals to the process of gene regulation. TFs can read the signals by binding to short regulatory sequences called transcription factor binding sites (TFBS) through their DNA binding domains (DBDs). DNA binding domains are structural motifs of protein domains recognizing single stranded or double stranded DNA. Position Weight Matrices (PWMs) or Position-specific scoring matrices (PSSMs) are frequently used for the representation of DNA-binding (TFDB) preference of transcription factors [1]. PWMs are obtained by aligning the experimentally validated TFBS while considering the number of occurrences and positions of the nucleotides in the binding sites. This model of representation of TFDB assumes that binding preferences are independent of each other. In some cases, there are dependencies between nucleotides [2, 3], which may need an alternative representation for TF binding. Nevertheless, PWMs are still the most popular representation of TFDB available. As the number of these TFDBs is increasing, several databases are maintained to keep this information such as JASPAR [4] and TRANSFAC [5] for experimentally derived TFDBs. These are the knowledge-bases used for interpretation of functional genomics results in DNA sequence analysis. Unfortunately, the increasing number of sources for PWMs has given rise to redundancy for TFDBs. For instance, different high throughput methods (e.g., protein binding microarrays, SELEX, ChIP-seq) generate different PSSMs or PWMs for the same TF. Apart from that, orthologues TFs from different organisms may also share similar TF binding profiles, as well as TFs from the same DBD family preserve similar binding specificity. These factors give rise to multiple TFs sharing similar binding motifs or PWMs. It has not only increased intra-databases redundancy, but also inflated inter-database redundancy. An exponential increase in the collection of PWMs (e.g., JASPAR [4] alone has doubled the number of its PWMs in just two years), and the generation of diverse PWMs from multiple studies [6, 7], may cause a problem in their application. For example, multiple collections of PWMs for the same TF may confuse people about using a trustful source and cause a problem in interpreting results based on different versions of PWMs. Although efforts have been made to solve the issue by creating meta-databases such as cis-BP [8] and FootprintDB [9], the redundancy problem in PWMs has not been fully addressed as yet.

Clustering of PWMs is an efficient solution to solve the redundancy problem. It has been used to cluster structurally similar motifs for making Familial Binding Profile (FBP) among related TFs. This has not only improved the performance of TF motif detection [10, 11], but also predicted DBD (or structural class) for a newly discovered motif [12] that has been utilized in a variety of functional genomic studies [13]. Although there are a handful of tools for clustering PWMs [14–17], none of them considers the DBD family information as well as the quality of clustering. A recently published tool MASSIF considers the DBD information, but it only evaluates the likelihood of a linked motif (or TF) of interest to be associated with the same DBD [18]. Since TFs are associated with different DBDs, a classification of TFs into respective DBDs may help in the downstream analysis. For example, if a large number of putative PWMs are predicted from a single high throughput sequencing (e.g., ChIP-seq and ATAC-seq) experiment, then they need to be clustered and quality evaluated before further biological interpretation of results [13]. Though numerous studies have attempted to classify TFs into relevant DBD families [4, 19, 20], none of them adopts any uniform naming convention. For example, transcription factor E47 is assigned to bHLH in some databases, but others define E2A as its DBD family, while E2A related factors is a subclass of bHLH [19]. Thus, there is a hierarchical mismatch between the assigned DBD name and the transcription factors. In addition, different nomenclature is used in different databases. For instance, the same TF is assigned to either bZIP or “Basic Leucine Zipper” in different sources, which is not suitable for automatic text mining. Some TFs do not completely function in a single DBD (e.g., E47 for bHLH [21]) and which require other protein regions for complete binding activity. Therefore, a uniformly named DBD database and a clustering quality assessment metrics for any given PWMs is urgently needed.

In order to meet the aforementioned challenges and fill the gap of quality assessment for clustering of PWMs, a new PWM analysis package was introduced—Affinity Based Clustering for Position Weight Matrices (abc4pwm). It included both PWMs clustering and quality evaluation. First, abc4pwm generates a clean uniform named DBD database from known human transcription factor classification databases [20, 22], assigns a set of known human TF PWMs into their respective DBD family, and clusters PWMs within each DBD family. Then, clustering quality assessment metrics are calculated in each cluster for filtering out wrongly clustered PWMs before further analysis (e.g., TF motif search). Abc4pwm has functions for visualization of PWMs clusters, and for searching a given PWM against known PWMs (e.g., from JASPAR or TRANSFAC) by reporting the top matched ones. It also has format conversion function for conversion between various formats (e.g., TRANSFAC [5], JASPAR[4], and BayesPI [23]). Moreover, the package also includes various demo applications of abc4pwm in DNA sequence analysis. For example, to predict TFs that are known for periodic regulation of yeast cell cycle by using gene expression profiles [24], to identify TF binding motif from ChIP-seq data or differentially expressed genes, and to perform ensemble clustering for PWMs predicted from ChIP-seq experiment [13, 25]. In short, Abc4pwm is a useful tool for DNA sequencings analysis which can reduce the analysis time and help in meaningful biological interpretations.

## Implementation

### Public data and tools

In abc4pwm package, ~ 1,770 human TF PWMs were collected from previous publications [26, 27]. DBD information for human transcription factors is retrieved from three sources (TFClass, The Human Transcription Factors by Jolma, and JASPAR) [4, 19, 20]. Yeast cell cycle microarray experiments of ~ 800 yeast cell cycle regulated genes were obtained from the Spellman study [28]. RNA-seq dataset for differential gene expression in HCT116 p53 +/+ cells (e.g., after inducing TP53 expression with Nutlin treatment compared to non-treatment) was downloaded from Andrysik et al. [29]. It contains ~ 4,363 differentially expressed genes, from which the up-regulated genes (~ 2,093) were used to predict putative TF binding motifs at promoters by using BayesPI2 [13, 23]. BayesPI2 is a tool that integrates a Bayesian model regularization method with biophysical modeling of protein-DNA interactions, to predict TF binding sites or PWMs at DNA sequences, from either promoter of genes or TF binding occupancy regions (e.g., ChIP-Seq). ER-alpha ChIP-Seq data in MCF7 cells was referred by Zhou et al. [30] and [13]. DNA sequences of gene promoter regions (i.e., − 800 and − 1000 to TSS in yeast and human data, respectively) were retrieved by the Regulatory Sequence Analysis Tool (RSAT) [31].

### Creation of uniform DBD database for human TFs

Here, a uniform DBD naming TF database in human was created for automatically assigning ~ 1,770 known human PWMs [26, 27] to their respective DBD family with a unique name. It was based on three sources, TFClass [19], The Human Transcription Factors by Jolma [20], and JASPAR [4], and automatically generated such non-redundant human TF DBD database by fetching information from these three online databases (Additional file 1: Figure S1). Based on this new DBD database, abc4pwm can assign any known human TFs to a respective DBD family. If a TF lacks DBD information, it was labeled as an unknown DBD category. PWMs assigned in each DBD family were further clustered by a clustering method.

### Pair-wise comparison of PWMs by similarity score

TF searching for any unknown PWMs was achieved by comparing them with known TF PWMs from existing databases. A previously published method forward–backward alignment [23] with motif similarity score calculation [32] (Additional file 1: methods) was implemented in abc4pwm to evaluate the similarity of PWMs. For example, a combination of similarity matrix calculation (n x n) for n input PWMs and a dynamical forward–backward PWM alignment method were used to perform a pair-wise comparison between PWMs. In this way, it not only generated a representative motif and evaluated the quality of PWMs in the same cluster, but also ranked top matched TFs after comparing unknown PWM with known sources (e.g., from TRANSFAC or JASPAR).

### Clustering of PWMs

As described earlier, there may be multiple PWMs for the same TF resulting from different computational or experimental studies. These PWMs from various sources may cause redundancy for data analysis. Clustering is thus a handy solution for such issue by grouping similar PWMs together, which significantly reduces the computation time and simplifies the data interpretation. However, prior information about the number of clusters for PWMs is usually absent, which is a challenge for unsupervised clustering methods. In such a case, many popular methods (e.g., K-means and DBSCAN clustering [33]) may not perform well because they need either an initial number of clusters or a cutoff value for grouping the clustering results. Affinity Propagation clustering instead requires only measures of similarity between pairs of input data points [34], then uses four matrices (similarity matrix—*S*, responsibility matrix—*R*, availability matrix—*A*, and criterion matrix—*C*) to perform unsupervised clustering. A similarity matrix *S* can be calculated from a pair-wise comparison between input PWMs with motif similarity score [23]. The remaining three matrices can be inferred from the similarity matrix. In abc4pwm package, this scalable clustering algorithm can be applied on either all input PWMs or PWMs in the same DBD family. The following is a short description of these four matrices in affinity propagation clustering:

- **Similarity matrix S**: A similarity matrix of dimension n x n, while n is the number of input data points or PWMs, is calculated for each data point against all input data. Values for diagonals are not calculated. The algorithm converges toward a small number of clusters if a smaller value is chosen for the diagonal, and vice versa. A formula for similarity matrix between two data points is given as:
  1 $$S\left(i,j\right)=-|\left|{X}_{i}-{X}_{j}\right|{|}^{2}$$

In abc4pwm, the similarity matrix is based on motif similarity score that described in previous section.

- **Responsibility matrix R**: Responsibility *R*(i, k) quantifies how well-suited element *k* is to be an exemplar for element *i*, which is calculated with the following formula:
  2 $$R(i, k)\leftarrow S(i, k) - \mathop{max \{A(i, k^{\prime}) +S(i, k^{\prime})\}}\limits_{k^{\prime}\,such\,that\,k^{\prime} \ne k}$$

where *A(i , k*) is availability matrix and *S(i , k)* is similarity matrix, and *A* is set to zero in the first iteration of calculation for *R.*

- **Availability matrix A**: From responsibility matrix *R*, algorithm calculates availability matrix. The availability matrix *A* shows how available one object or data point is to be an exemplar for another object or data point. Calculation for diagonal data points can be computed as:
  3 $$A(k, k) \leftarrow \mathop{\Sigma\mathrm{ max }\{0, R(i^{\prime}, k)\}}\limits_{i^{\prime}\,such\,that\,i^{\prime} \ne k}$$

While off-diagonal points can be calculated as:

4 $$A(i, k)\leftarrow \mathop{\mathrm{min}\{0,R\left(k,k\right)+\Sigma \mathrm{max}\left\{0, R\left({i}^{^{\prime}},k\right)\right\}\}}\limits_{{i}^{{{\prime}}}such\,that\,{i}{{^{\prime}}}\notin \{i,k\}}$$

where *R* refers to the responsibility matrix.

- **Criterion Matrix C**: The last matrix is named as criterion matrix *C*. Each cell in the criterion matrix is the sum of the responsibility matrix *R* and availability matrix *A* at that index.
  5 $$C (i, k) \leftarrow R(i, k) + A(i, k )$$

A pseudo code for Affinity Propagation Clustering Algorithm based on aforementioned Eqs. 2, 3, 4, and 5 was provided in (Additional file 1: methods), where more information about ideas behind Eqs. 2, 3 and 4 were illustrated, as well as a step-by-step example of calculations was provided. Two parameters (e.g., preference and damping) associated with affinity propagation clustering may need to be tuned during the training (see the Additional file 1: method). In abc4pwm, PWMs with known DBD information can be classified into their respective DBDs before the clustering. Alternatively, clustering of all available PWMs can be performed at once though it may not provide good clustering quality (e.g., in ~ 1,770 human TFs PWMs) [14].

### Clustering quality assessment and representative motif for PWMs

**Fig. 1:**
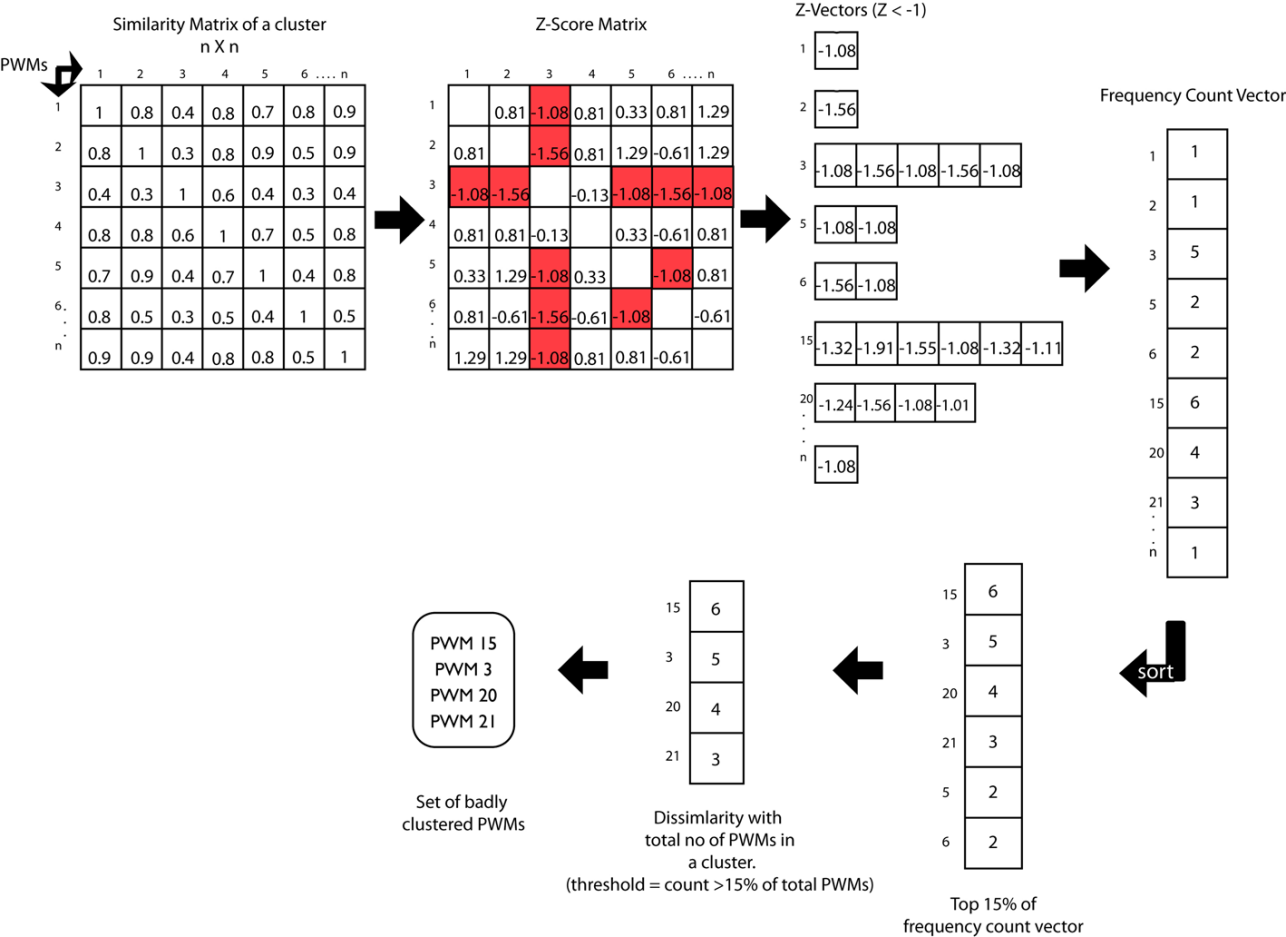
Automatic quality assessment method for PWM clustering. First, a similarity score matrix for PWMs in a cluster is calculated and Z-score is calculated for each row (one row represents one PWM; Z-scores of one PWM versus all others). Then, Z-scores less than a threshold (e.g., < − 1) are counted to make a frequency count vector, which is sorted and the top 15% of them (default parameter in abc4pwm) are selected as putative poorly clustered PWMs. Finally, the poorly clustered PWMs are identified and be removed from clusters (e.g., PWMs 15, 3, 20 and 21 in the figure)

**Fig. 2:**
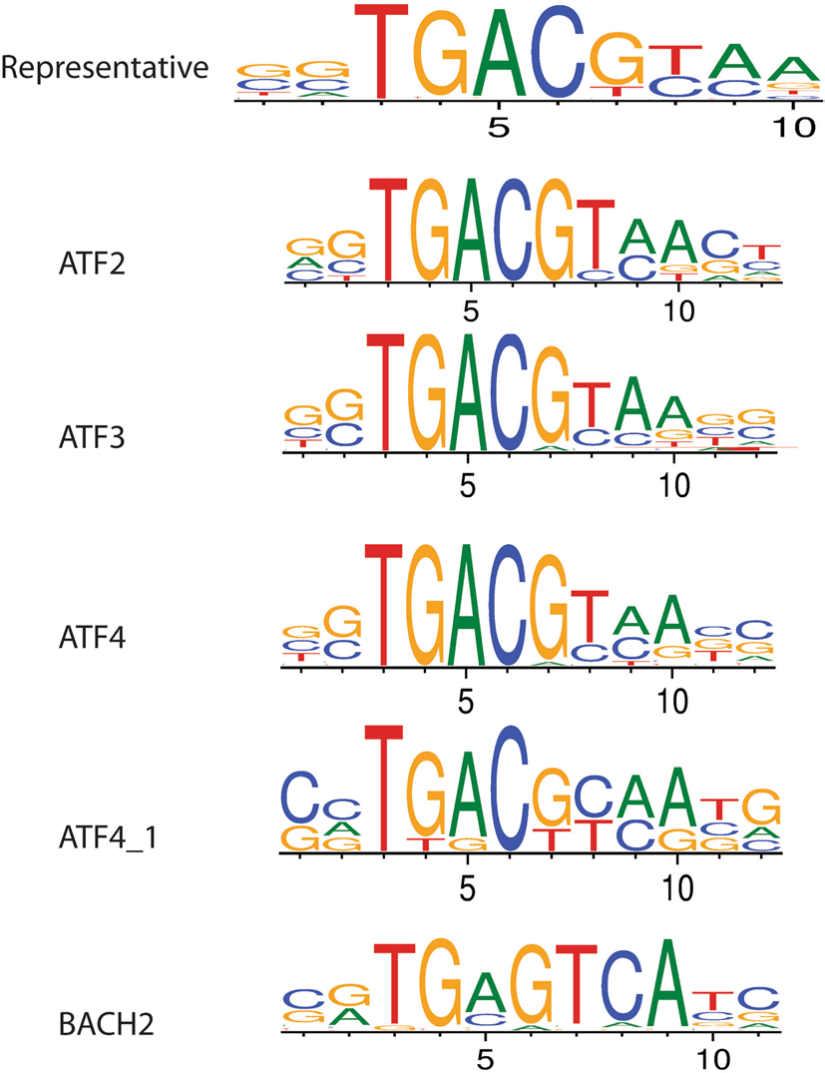
An example of representative motif for a cluster of PWMs in bZIP DBD family. Here, there are five PWMs in a cluster (ATF2, ATF3, ATF4, ATF4_1 and BACH2) from bZIP family. A representative motif of this cluster is shown on the top of figure

Quality assessment for clustered PWMs is essential, but there is only one feature (a similarity score matrix for all input PWMs) in the current study. Therefore, a combination of several statistical summaries was used in the quality assessment of clustered PWMs (e.g., mean and Z-score of similarity scores in a cluster). In a good cluster, if the mean of similarity scores for PWMS is higher (e.g., >  = 0.8 default parameter) then the PWMs are more similar. After identifying the good clusters, the poorly grouped PWMs was further evaluated. Here, a Z-score was used to indicate how different a PWM is from the mean of the cluster: a Z-score matrix (n x n) of PWM similarity scores in a cluster was calculated for each PWM. Any PWM with Z-score < − 1 (default parameter) will be subjected to further evaluation, and the frequency of PWMs with low similarity scores (e.g., Z-score < − 1) in a cluster will be recorded. Then, the top 15% (default parameter) of such PWMs were defined as poorly clustered PWMs. Finally, PWMs with poor quality were removed from a cluster (e.g., poor similarity scores with Z-score < − 1 and has at least 5% of other PWMs in the cluster). A detailed graphical overview of such cluster quality assessment for PWMs was shown in Fig. 1. After the automatic clustering quality assessment process, a representative PWM (or motif) was generated [13] for each cluster which was an average of all PWMs (Fig. 2) in the same cluster.

### Motif discovery using ensemble learning approach

abc4pwm implemented a new ensemble learning approach to predict PWMs from protein-DNA interaction experiments (e.g., ChIP-seq or ChIP-chip) [13, 23]. First, it randomly selected a subset of input data (e.g., 15% of called peaks from a ChIP-Seq experiment) for predicting enriched TF binding energy matrices (or PWMs) in DNA sequences, by using BayesPI2[13]. The random selection and the PWM prediction were repeated multiple times (e.g., 10 times by default), before PWMs were clustered and the quality was assessed. Subsequently, the representative motifs were generated for the clusters and were compared against known TF PWMs, from which the top matched ones are reported. In abc4pwm, a parallel implementation of the ensemble learning approach for PWM clustering was adapted from a previously publication [13]. Multiple parameters were available for customization which can be easily tuned to improve this analysis (e.g., the random seed number, the percentage of input data, the number of random selections, the maximum/minimum length of motifs, and the on/off of clustering quality assessment et al.). The ensemble learning approach significantly speeds up the motif discovery process, if the input is large (e.g., hundred thousand of called peaks from a ChIP-Seq experiment). It has been evaluated in both synthetic yeast ChIP-chip and real human TF ChIP-seq datasets (e.g., the estrogen receptor α factor—ESR1).

### Modules in abc4pwm

**Fig. 3:**
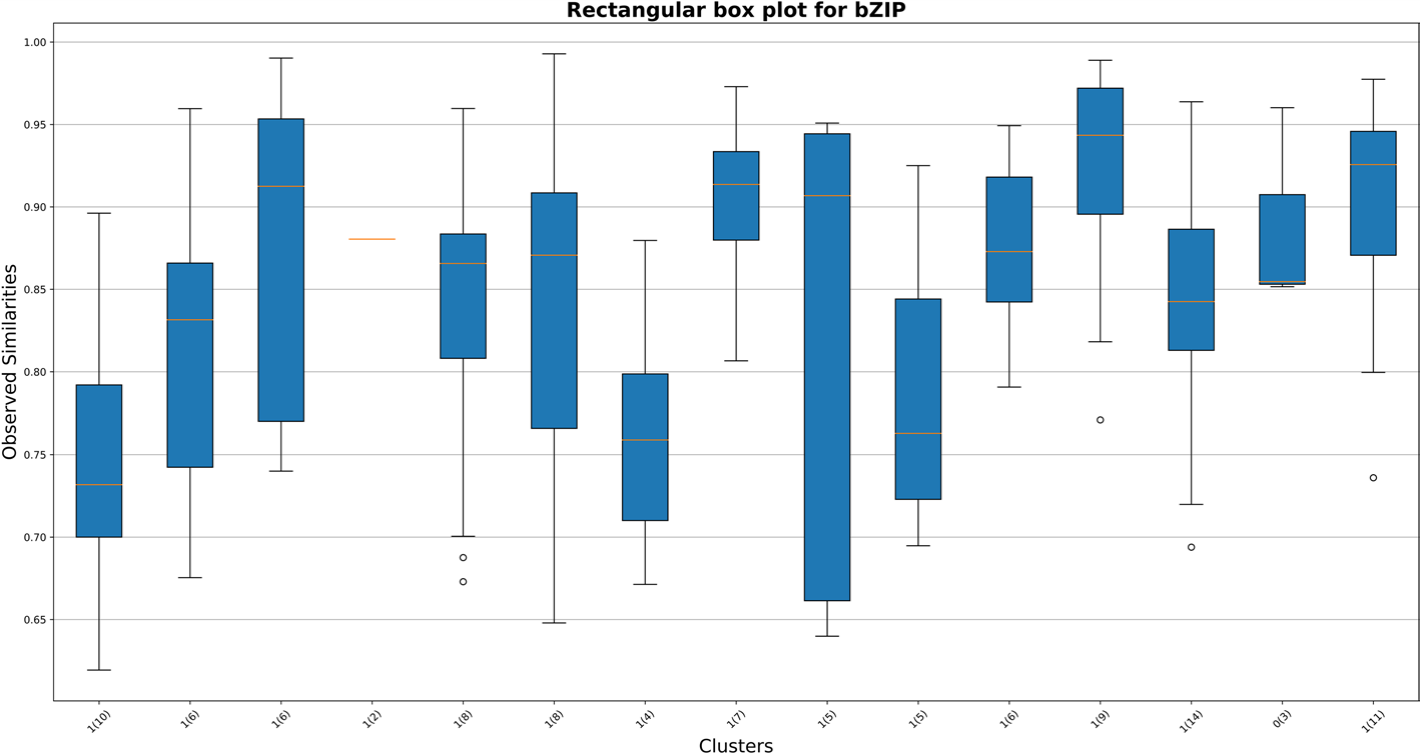
A boxplot of similarity scores for PWMs in clusters of bZIP DBD family*.* This figure shows 15 PWM clusters in a bZIP DBD. X-axis indicates the number of poorly clustered PWMs (total number of PWMs) in each cluster. Y-axis shows the distribution of PWM similarity scores in each cluster

**Fig. 4:**
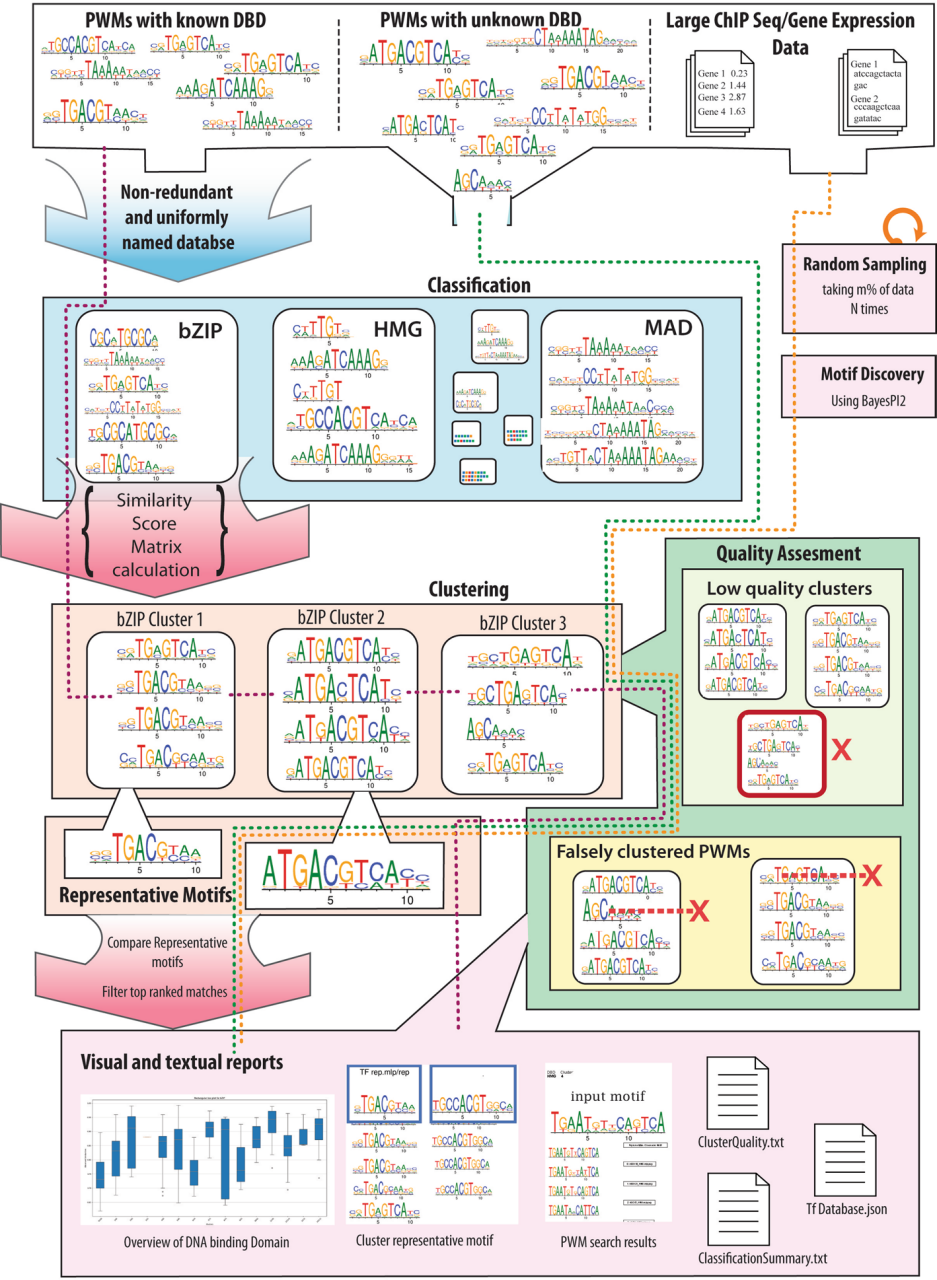
An overview of abc4pwm workflow. This figure shows an overview of all major features in abc4pwm. Purple line flow shows classification module where input PWMs are divided into DBD. Then, clustering module (orange flow) is applied within each DBD. Subsequently, resulted clusters are subjected to quality assessment (green flow) and a representative motif or PWM is created for each cluster. Green dotted line shows flow where input PWMs skip DBD assignment step. Orange dotted line shows the flow of ensemble learning technique for motif prediction

Abc4pwm was implemented in various features modules, including plotting, visualizing, and searching etc. For example, a “plotting” module to visualize PWM clustering with weblogo [35]. It not only plots all clusters in a specified DBD, but also plots all clusters for all DBDs. Results of ‘plotting’ module are exported to both a pdf file and a text file. Another module ‘visualize’ generates statistical summary figures for the clustering quality assessment (e.g., boxplots of similarity scores for PWMs of clusters in a DBD family; Fig. 3) at any desired DBD family. The other important feature is “searching” that compares unknown PWMs to a list (or database) of known TF PWMs (e.g., from TRANSFAC [5], JASPAR [4], abc4pwm, or BayesPI [23]), and reports the top matched ones. Here, Logo plots of all query PWMs and the top matched ones are exported to a pdf file. Other results (e.g., the similarity scores, DBD information, and cluster number) and parameters (e.g., information of matched DBD source, DBD family labels, the number of PWMs/clusters in a DBD, and clustering parameters etc.) are stored in text files. A full illustration of functions in abc4pwm was shown in Fig. 4. abc4pwm is a command line tool in Python 3.0, which is suitable for analyzing large DNA sequencing data with parallel computation in either PCs or high-performance computing clusters (HPCs). It is publicly available at (https://github.com/abc4pwm/abc4pwm) and can be easily installed on both Linux and Mac OS systems.

## Results and discussion

### Clustering quality assessment: manual versus automatic evaluation

**Fig. 5:**
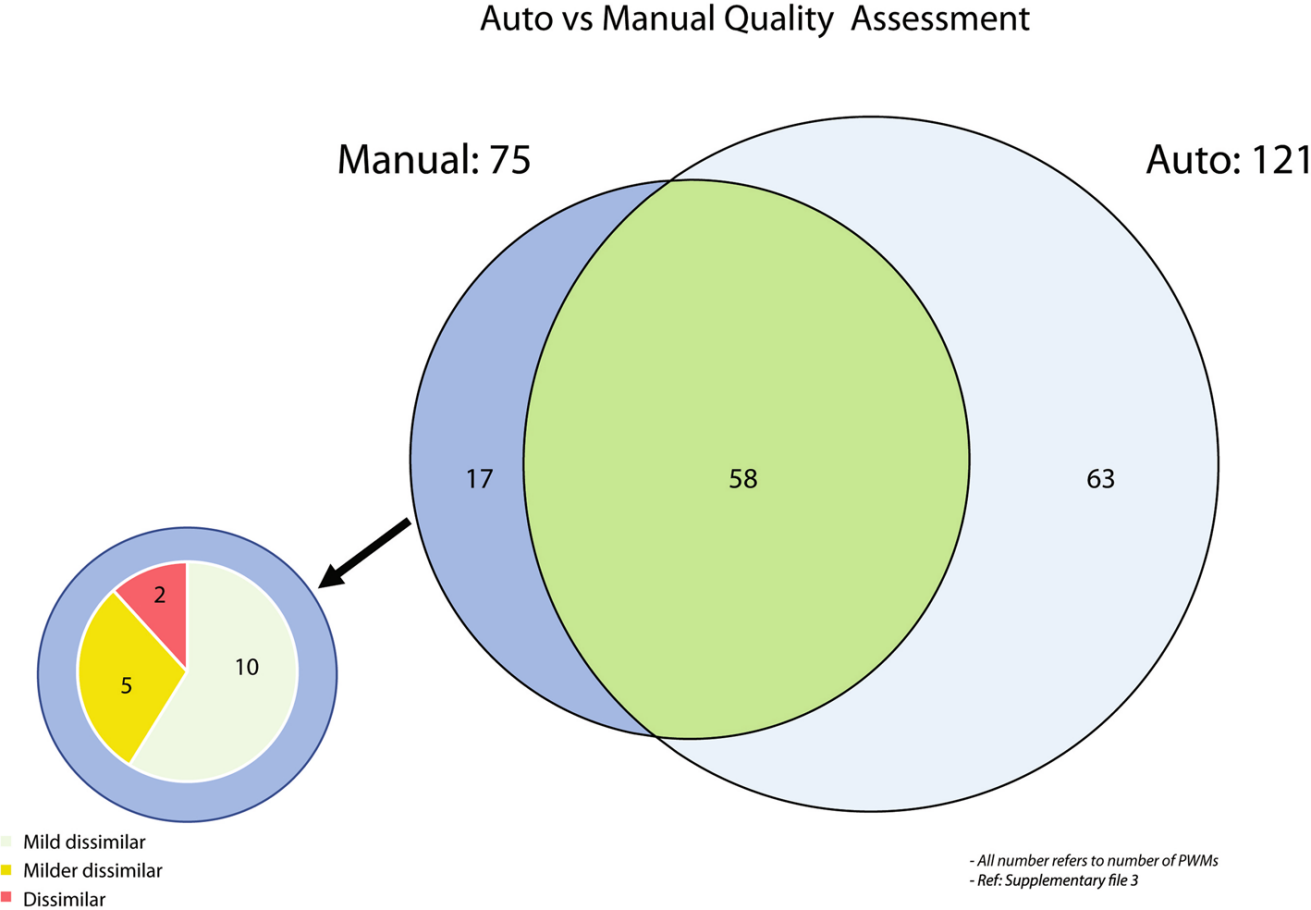
Comparison between automatic quality assessment and manual quality assessment. Here, dark blue represents manual or eye balling assessment of quality for clustered PWMs, where 75 PWMs were identified as poorly clustered. Light blue color shows result of automatic quality assessment for the same clusters provided by abc4pwm, where 121 PWMs were marked as poor quality. There are 58 of PWMs overlapping (dark green color) between the two results. The remaining 15 out of 17 that identified by manual evaluation have mild dissimilarity

A collection of ~ 1,770 human TF PWMs [36] was used to evaluate the automatic clustering quality assessment in abc4pwm. First, a clean and uniformly named DBD database was generated to maintain human TF DBD information from three sources (TFClass [22], Human Transcription Factors [20], and JASPAR [4]). Then, these human TF PWMs were classified into their respective DBD family based on the new DBD database. Within each DBD family, PWMs were clustered and the quality of clusters was automatically evaluated by abc4pwm. In this analysis, there were ~ 121 poorly clustered PWMs (mean, Z-score, the top occurrences, and the occurrences threshold in abc4pwm was set 0.80, − 1.2, 0.05, and 0.05, respectively). Based on a manual examination of the quality of the same clusters, there were 75 wrongly clustered PWMs. Among them, ~ 77% (58 out of 75) were labeled by the automatic assessment as wrongly clustered (Fig. 5). In the figure, a “mild dissimilar” refers to PWMs with 40–50% non-identical nucleotides, while a “milder dissimilar” refers to 60–70% non-identical nucleotides in a PWM. If a less stringent criteria (e.g., PWMs with ~ 50% identical nucleotides are considered as similar) was used in manual quality assessment, then 73 out of 75 wrongly clustered PWMs were found by both the manual and automatic evaluations. This was a very encouraging result because there was a large overlap for clustering quality assessment between the manual and the automatic clustering evaluation. A detailed description of this comparison was shown in Additional file 2, which showed that the automatic clustering quality assessment worked well in general, but it did not mean the automatic quality assessment would always be better than the manual examination. Nevertheless, the automatic clustering quality assessment in abc4pwm was a robust method, which may significantly reduce the time for evaluation and interpretation of large numbers of PWMs.

### Clustering of PWMs with-DBD versus no-DBD information

**Fig. 6:**
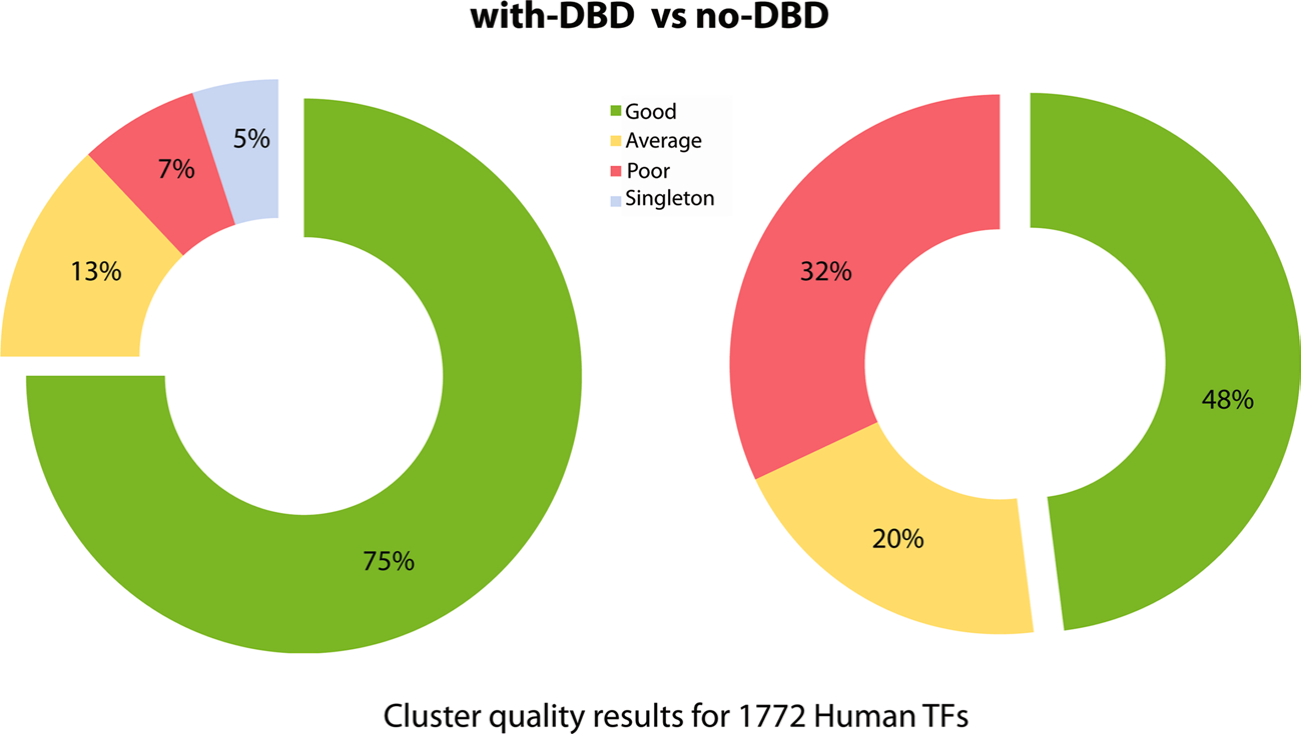
Comparison clustering quality of PWMs between with and no DBD information. Here, green color represents good homogeneous clusters, yellow means average quality clusters, red are bad quality ones (clusters without similar PWMs). Left panel shows clustering results of TF PWMs by classifying them to DBD family before clustering (with-DBD), while right panel shows clustering results for the same set of PWMs by clustering them directly without consider DBD information (no-DBD)

Initially, clustering of PWMs was applied on ~ 1,770 collected Human TF PWMs directly (no-DBD information) where many PWMs were wrongly clustered (right panel of Fig. 6). In the figure, green and yellow colors represent good quality (~ 48%) and average quality clusters (~ 20%), respectively. The good quality clusters are those with ~ 85–100% of similar PWMs, the average and the bad ones are clusters with ~ 50–80% and < 50% similar PWMs, respectively. In summary, there were ~ 32% of the clusters with poor quality in no-DBD clustering. However, classifying PWMs into relevant DBD family (with-DBD information) and then clustering them within each DBD family had significantly improved the results as shown in the left panel of Fig. 6. The total number of good quality clusters (~ 75%; green color) increased and average quality (~ 13%; yellow color) clusters are decreased. And also, there were only ~ 7% and ~ 5% of the poor-quality clusters and singletons (clusters with only one PWM), respectively. Thus, clustering PWMs with-DBD information provided better results than that with no-DBD information. Detailed supporting data of this summary were in Additional files 3 and 4.

### Clustering of PWMs: abc4pwm versus STAMP

To further evaluate the performance of PWM clustering by abc4pwm, we compared it with one of the popular tools, STAMP [16] that generates a similarity score tree to cluster PWMs. From a collection of ~ 1770 human PWMs, we selected PWMs from six DBD families (e.g., HMG, C2H2_ZF, Tryptophan, T_box, RHR, and bSHS), converted them to TRANSFAC format, then submitted these PWMs to STAMP online tool. These six DBD families include small (~ 3–4 clusters e.g., RHR, bSHS, T_box), average (~ 11–12 clusters e.g., HMG, Tryptophan), and large (~ 20–25 clusters e.g., C2H2_ZF) size of DBDs. For STAMP, a column comparison metric Pearson Correlation Coefficient, un-gapped Smith Waterman alignment method, and UPGMA were chosen for Tree building algorithm. Tree results were visualized by MEGAX [37] and kept to have similar number of clusters between STAMP and abc4pwm. For abc4pwm, default parameters were used in clustering of PWMs. A comparison of clustering results between the two methods was assessed by manual assessment. For small DBD family, three good PWM clusters were produced by both tools in RHR; six and five good clusters were predicted by abc4pwm and STAMP in T-box, respectively; four good clusters were obtained by both tools in bSHS family. For average sized DBDs, 11 clusters were obtained by both tools in HMG, where 10 and 9 of them were of good quality from abc4pwm and STAMP, respectively; 12 and 10 clusters were obtained from both tools in Tryptophan, where 12 and 7 good clusters from abc4pwm and STAMP, respectively. For large sized DBD, 24 and 18 clusters were produced in C2H2_ZF by the two tools, respectively, where 12 and 6 were good clusters but 5 and 9 bad ones. More information of this comparison is illustrated in Table 1. These results indicate that both tools can produce PWM clusters with good quality for small sized DBDs, but abc4pwm provide better clusters than STAMP for average and large sized DBDs. Thus, abc4pwm is a robust tool for clustering large number of PWMs.

**Table 1:** A comparison of PWM clusters between abc4pwm and STAMP

DBD family	PWMs in DBD	Abc4pwm	STAMP
RHR	20	All good (3)	All good (3)
bHSH	27	All good (4)	1 mix 3 good (4)
T_box	32	All good (6)	All good (5)
Tryptophan	95	All good (12)	2 bad 1 mix 7 good (10)
HMG	101	10 good 1 mix (11)	2 bad, 9 good (11)
C2H2_ZF	161	5 bad 7 mix 12 good (24)	9 bad 3 mix 6 good (18)

Here, results of a manual comparison of clustering quality between abc4pwm and STAMP are provided. Good = more than 90% of the PWMs in a cluster are identical, Mix = 70% of the PWMs in a cluster are similar, and Bad = less than 30% of the PWMs in a cluster are similar. Total number of clusters in each DBD family are shown in the parenthesis

### Application of abc4pwm in predicting yeast cell cycle transcription factors by using gene expression profiles

The cell cycle is regulated by complex transcriptional programs in cells which is periodically self-regulated through an intricate interplay between trans-acting elements and their respective regulated genes. The yeast cell cycle is known to be regulated by three types of transcription factors; MBF and SBF factors, Mcm1p-containing factors, and Swi5p/Ace2p. In total, there are nine TFs known to regulate the yeast cell cycle: Ace2,FKH1, FKH2, MBP1, MCM1, NDD, SWI4, SWI5, SWI6 [38]. Spellman *et.al.* identified a comprehensive list of ~ 800 genes that are periodically regulated in the yeast cell cycle by the aforementioned set of transcription factors [28]. Here, BayesPI2 [13, 23] was used to predict protein binding energy matrices (PBEMs) enriched in the upstream regulatory region of genes, by using microarray experiments of the putative 800 yeast cell cycle regulated genes from the Spellman study [28]. Then, abc4pwm was used to compare the predicted PBEMs against the known PWMs of the desired yeast TFs.

Alpha pheromone arrest was used in the aforementioned study to arrest cell cycle at different stages [27]. Three time points (alpha 7, alpha 42 and alpha 49) that correspond to M/G1 and S/G2 of the yeast cell cycle were used in this study. Unlike higher eukaryotes where regulatory sequences for transcription are located anywhere in the noncoding genome, in case of yeast they are located upstream of transcription start site [39]. In light of aforementioned information and the previously published study [24] 800 bp upstream of the genes was chosen to search for TF binding motifs and their energy matrices. From which, multiple PBEMs were predicted by BayesPI2 for motif length ranging between 6 and 14 bps on both reverse and forward strands. Subsequently, all the predicted PBEMs, for each time point, were searched against the database of PWMs of nine yeast TFs by using abc4pwm. For the time point alpha7, almost all nine TFs (SWI4, SWI5, MCM1, NDD1, ACE2, MBP1, FKH1) have their binding sites predicted as shown in Additional file 1: Table S1. It fits with previous observation in ChIP-chip [38] that M/G1 cell cycle phase has binding preference for almost nine yeast cell cycle related TFs [38]. Results, were presented in Additional file 1 of Figure S2A. Alpha 42 and 49 were the timepoints adjacent to each other (S/G2 phase) which means the set of transcription factors with active binding sites during these two time points must be overlapping. Abc4pwm predicted binding sites for SWI4, NDD1, MBP1and MCM1for alpha 42 (Additional file 1: Table S2). For alpha49, NDD1 and ACE2 binding sites were found highly enriched (Additional file 1: Figure S2B, Table S3). Alpha7 depicts arrest in M/G1 phase, while Alpha42 and 49 depict arrest in S/G2 phase. M and G1 phase of yeast cell cycle involved most of the TFs (Ace2, FKH1, FKH2, MBP1, MCM1, SWI4, SWI5, SWI6) with different intensities. Using abc4pwm, we were able to report PBEMs for 7 out of 8 of these TFs correctly. On the other hand, S/G2 phase requires involvement of FKH1, FKH2, NDD1, MCM1, and SWI4. The abc4pwm recovered 3 out of 5 of TFs with accuracy. However, it has been reported that Fkh1/Fkh2 are required to recruit NDD1 in G2 phase. Absence of Fkh1/Fkh2 from prediction by abc4pwm might be due to intermediate roles of these TFs at this stage of cell cycle. Logo plots and similarity score predicted by abc4pwm for these PWMs are shown in Additional file 1 (Tables S1, S2, and S3) and demo for yeast cell cycle in abc4pwm package.

### Application of abc4pwm in motif prediction for ESR1 ChIP-Seq data

**Fig. 7:**
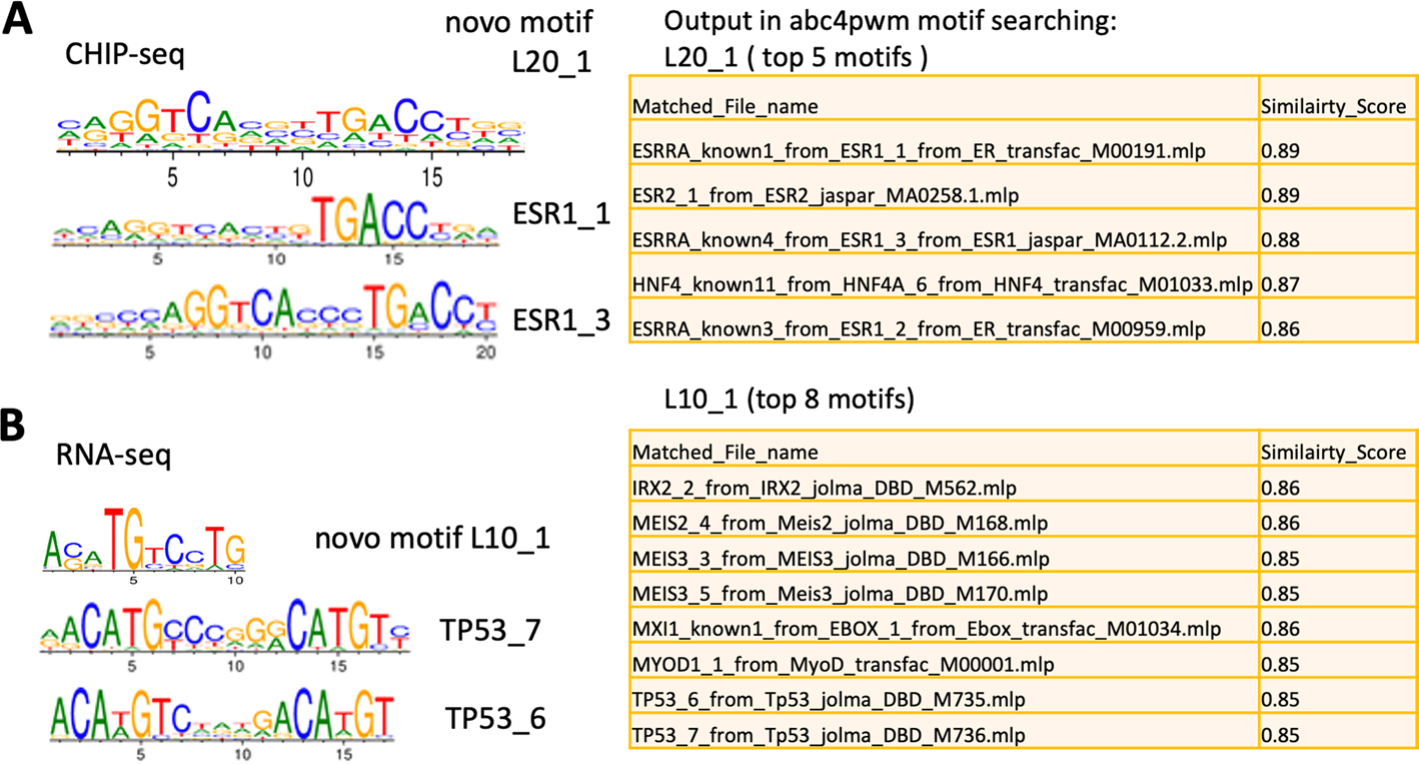
Application of abc4pwm in TF binding motif prediction by using either ChIP-seq data or gene expression profiles. **A** Application of abc4pwm in ESR1 CHIP-seq data in MCF7 cells, where the predicted novo motif L20_1 is similar to known-ESR1-1,2,3 motifs (similarity score = 0.89) based on motif search module of abc4pwm. **B** Application of abc4pwm in RNA-seq data of TP-53 knockout experiment, the top enriched novo motif L10_1 in promoters of differentially expressed genes is similar to known-TP53 motifs TP53_6 and TP53_7 (similarity score = 0.85). The left panel is the motif logo of TFs, the right panel shows the output of motif similar scores from abc4pwm searching module

To explore the application of abc4pwm package in motif prediction, we evaluated its ability in finding true TF targets for motifs predicted from ChIP-Seq experiment. The estrogen receptor alpha (ESR1) is an important transcriptional regulator, known to mediate the effects of estrogen mainly by binding to the conserved motifs of the targeted genes in their promoter regions [30]. We extracted the reads count (called peak abundance) and the related sequences from ESR1ChIP-seq experiment in human MCF7 cells, which were used as input data for BayesPI2. It predicted three highly enriched motifs or PBEMs of 20 bp. Then, abc4pwm search module was used to compare these predicted PBEMs against ~ 1,770 collected human TF PWMs. Among top ranked TFs, one of the enriched motifs from ChIP-seq data was similar to 3 known-ESR1 PWMs (e.g., with similarity score ranging between 0.89 and 0.86; Fig. 7A). It suggests that abc4pwm is a useful tool to identify true target TFs for de novo motifs predicted from in vivo ChIP-seq experiments.

### Application of abc4pwm in TP53 binding motif prediction in RNA-seq dataset

Here, abc4pwm package was further evaluated to identify de novo motifs in RNA-seq datasets. RNA-seq is widely used to identify differentially expressed genes (DEGs) in transcriptome, DEGs enrichment in biological pathways and in molecular functions. To identify the conserved motifs or motifs enrichments among the DEGs is crucial for further understanding the transcription networks and it has great potential to find the key regulators in disease conditions or after drug/therapy treatments. Currently, only few packages were available to search motifs from RNA-seq dataset, such as HOMER [40]. Here we used BayesPI2 to predict novo motifs from DEGs based on promoter sequence and transcription in fold change level, and further used abc4pwm to search for true TF targets of these motifs (e.g., similarity search against known motifs). A previously published RNA-seq dataset from HCT116 p53 +/+ cells after inducing TP53 expression with Nutlin treatment [29] was used in this work. TP53 is a crucial transcription factor, binding to at least 542 binding loci via a conserved motif. It plays key role in p53-dependent tumorigenesis in primary cancer [41]. The consensus binding site of the TP53 motif consists of two copies of the 10 base pair motif 5′-PuPuPuC(A/T)(T/A)GPyPyPy-3′ separated by variable base pairs [42].

In the RNA-seq dataset, there were ~ 2,093 up-regulated genes induced by drug treatment compared to non-treatment. A main proportion of these genes were TP53 targets in transcriptional regulation. First, the promoter region 1,000 bp upstream of TSS of each DEG were retrieved using RSAT tool. The novo motifs inside the promoter region were identified by BayesPI2. By searching for a collection of ~ 1770 human TF PWMs in abc4pwm, a set of top ranked the novo motifs (e.g., Fig. 7B, 9–12 bp length, similarity scores 0.80–0.85) were found to be similar with four known TP53 motifs as well as the consensus binding site in literature [41]. For example, the novo motif L10_1 was found similar to two known TP53 motifs in the top 8 ranked motifs, which was also similar to motifs of IRX2, Meis2-3 and EBOX1. These results showed that TP53 motif was successfully recovered from DEGs in RNA-seq dataset, supporting that the TP53 binding to its conserved motifs was the driving force in the regulatory network after Nutlin treatment. Thus, by using both motif prediction tool and abc4pwm package, we were able to discover putative target TFs that regulate gene expressions in microarray or RNA-seq experiments, as well as to identify potential target genes of TF through in vivo protein-DNA interaction measurements such as ChIP-seq.

### Ensemble learning of PWM clustering in synthetic and real ChIP-Seq data

**Fig. 8:**
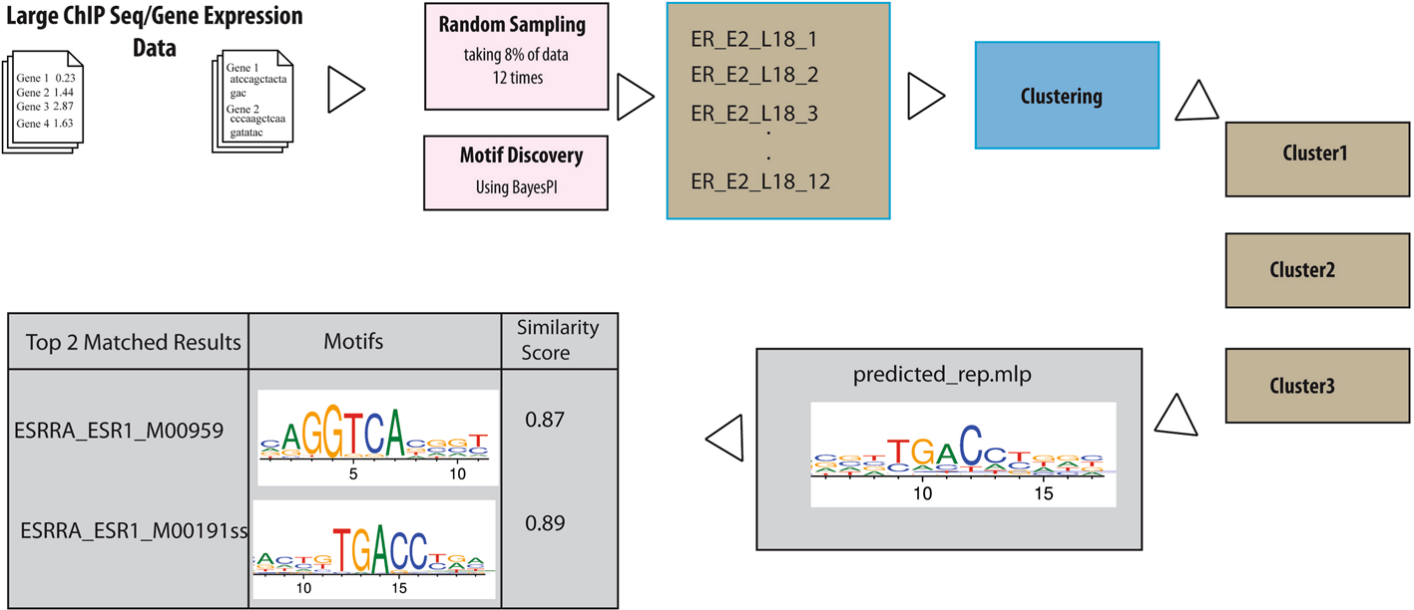
Applying an ensemble learning approach to predict TF binding motifs from ESR1 ChIP-seq data. First, input data is randomly selected multiple times from all called peaks from ESR1 ChIP-seq experiment in MCF7 cell line for predicting enriched motif, by using bayesPI2. Then, all predicted PWMs from multiple selections are clustered and quality evaluated by abc4pwm (e.g., three clusters indicated by brown color). Representative motifs or PWMs of good quality clusters are generated, and are used to search against known PWMs of human TFs (~ 1770 PWMs) by using searching module of abc4pwm (gray colored box). The top two matched search results (ESR1_M00959 and ESR1_M00191) are displayed along with their similarity scores, where the motif images are cropped to highlight matched areas

ChIP-chip and ChIP-seq are widely used for in vivo protein-DNA interaction studies. One of the essential tasks is to identify TF binding sites in the regulatory regions. A new ensemble learning approach for PWM clustering [23] was applied on yeast SWI4 ChIP-chip synthetic dataset. The predicted PWMs were then clustered, quality evaluated, and searched against ~ 233 known yeast PWMs from SGD database. As expected, the top matched yeast TF is SWI4 in this test (i.e., similarity score > 0.80). Results and demo script file for this analysis is included in abc4pwm package. Subsequently, the same procedure was applied on ESR1 ChIP-seq data in MCF7 cell lines [43], where 10 times randomly selected (e.g., ~ 8%) called peaks (~ 16,500) were used to predict enriched PWMs (motif length from 16 to 22 bp). All predicted PWMs were clustered into 33 clusters, and 4 of them passed clustering quality assessment in abc4pwm. Then, representative motifs of these good quality clusters were generated and searched against known human PWMs (i.e., a collection of ~ 1770 human TF PWMs). The top 2 matched human TFs were reported for each cluster (e.g., similarity score > 0.80), and representative motifs match to two human ESR1/ESRRA PWMs are shown in Fig. 8. Results and scripts of this analysis are also included in abc4pwm package. Thus, the ensemble learning approach in abc4pwm not only improves the computational efficiency for large input data (> 10,000 called peaks), but also predicts the correct TF for in vivo ChIP-seq experiment by searching for a large number of known human PWMs. More details of the ensemble learning for DNA motif analysis is available in the demo of abc4pwm package.

## Conclusions

Abc4pwm is a command line Python software package for TF analysis, which can cluster large sets of PWMs like other published tools [17, 44]. However, the better clustering results and the novel clustering features in the package, such as the automatic quality assessment for clustered PWMs and the ensemble learning approach for motif enrichment prediction, make abc4pwm an unique tool for DNA sequence analysis. In this work, biological applications of abc4pwm were illustrated in both synthetic and real experimental datasets. For example, abc4pwm successfully clustered ~ 1,770 human TFs to ~ 271 clusters by considering DBD information, predicted correct PWMs for yeast cell cycle TFs and human TP53 TF from gene expression profiles, and identified true TF binding sites from ER-alpha ChIP-seq experiment in MCF7 cells. All of aforementioned demos were included in the package, which can be a useful resource for biologists to perform DNA sequence analysis (e.g., in gene expression profile, ChIP-chip or ChIP-seq data) for understanding complex gene regulatory networks. For instance, the searching module of abc4pwm can associate the newly discovered TFBS with known TFs. Both the classification of PWMs to DBD family and the clustering of PWMs modules help generate familial binding profiles for PWMs in the same cluster, which significantly reduces the computational and interpretation time of further data analysis. In addition, abc4pwm includes a novel method for automatic PWM clustering quality assessment, which simplifies the cluster quality evaluation and expedites the further biological interpretation of data. Abc4pwm is easy to be installed and utilized by any user with basic knowledge of computers. Package is tested and operational for variety of platforms i.e. Mac OS and Linux system. The tool was implemented in parallel computations to optimize most of calculations and is scalable for large input data. Hence, it is suitable for small scale computations on personal computers as well as for large scale computations on high performance computing systems. In conclusion, abc4pwm is not only useful for clustering of PWMs, but also for the association of unknown PWMs to known regulatory elements in the genome. It can help researchers in DNA sequence analysis for exploring various regulatory elements in the genome.

## Availability and requirements

Abc4pwm is available under the MIT license from: https://github.com/abc4pwm/abc4pwm. A README file is included for comprehensive description of its features. The datasets used and/or analyzed during the current study are available from the corresponding references.

- Project name: abc4pwm
- Project home page: https://omer0191.github.io/abc4pwm/
- Operating system(s): Mac or Linux
- Programming language: Python
- License: MIT License
- Any restrictions to use by non-academics: None

## Supplementary Information

**Additional file 1:** Supplementary methods and figures. The file contains supplementary methods, figures and tables related to the main text.

**Additional file 2:** Manual versus automatic clustering quality evaluation. The file contains results of comparison between manual and automatic clusters quality evaluation in each DBD family.

**Additional file 3:** Manual evaluation of cluster quality for clustering within each DBD family. The file contains results for details of manual evaluation of clustering quality in each DBD family.

**Additional file 4:** Manual evaluation of cluster quality for clustering 1772 PWMs. The file contains results for manual evaluation of clustering quality for 1772 PWMs without DBD information.

## Data Availability

All datasets used to illustrate abc4pwm functionalities were previously published and are cited in the text. Datasets used to generate Figs. 7 and 8 were obtained from the NCBI Gene Expression Omnibus, IDs: GSE86222 and GSE14664, respectively.

## Abbreviations

TF: Transcription factor
DBD: DNA-binding domain
PWM: Position weight matrices
