# Supplementary material for: abc4pwm: affinity based clustering for position weight matrices in applications of DNA sequence analysis
Source: BMC Bioinformatics. 2022 Mar 3;23:83. doi: 10.1186/s12859-022-04615-z (PMC8896320; doi:10.1186/s12859-022-04615-z)
Supplement: Supplementary file 1 — Additional file 1: Supplementary methods and figures. The file contains supplementary methods, figures and tables related to the main text. [file 12859_2022_4615_MOESM1_ESM.docx]

**ABC4PWM: Affinity Based Clustering for Position Weight Matrices in Applications of DNA Sequence Analysis**

**Omer Ali ^1^, Amna Farooq ^1^, Mingyi Yang ^3,4^, Victor Jin^5^, Magnar Bjørås ^4,7^, Junbai Wang ^2,6*^**

1. Department of Pathology, Oslo University Hospital - Norwegian Radium Hospital, Oslo, Norway
2. Department of Clinical Molecular Biology, Institute of Clinical Medicine, University of Oslo, Norway
3. Department of Medical Biochemistry, Oslo University Hospital and University of Oslo, Oslo, Norway
4. Department of Microbiology, Oslo University Hospital and University of Oslo, Oslo, Norway.

5. Department of Molecular Medicine, University of Texas Health San Antonio, San Antonio, TX, USA

6. Department of clinical molecular biology (EpiGen), Akershus University Hospital, Lørenskog, Norway

. 7. Department of Clinical and Molecular Medicine, Norwegian University of Science and Technology, Trondheim, Norway

**Supplementary Methods:**

**Pair-wise comparison of PWMs by using motif similarity score**

To compare the similarity of two PWMs (e.g., between a predicted PWM and a known sequence specificity PWM from an existing database), and/or to evaluate the quality of clustered PWMs within the same cluster, a method to evaluate similarity between a pair of PWMs is required. Hence, we use a published strategy to accomplish this goal, a similarity between two PWMs is calculated by first converting them into position specific probability matrix (probabilities of nucleotide *i* at position *j* in the position weight matrix [1])

$$P\left( i,j \right)=\frac{f_{i,j}+P_{i}}{k+1}$$

where$f_{i,j}$is the frequency of residue *i* at position *j*, $P_{i}$is the prior frequency for residue *i,* and k is the number of k-mers. Similarity score is then calculated by considering the maximum overlap and maximum score between a pair of matrices during the alignment, where around 10% of misalignment between the two position probability matrices is allowed and both forward and reversed DNA sequence strands are considered. Here the highest similarity score is selected as the final maximum similarity score between the two matrices. It is similar to a previously published method [2]

$$\mathrm{similarit}y_{\mathrm{score}}=1-\frac{1}{w}\sum_{i=1}^{m} \frac{1}{\sqrt{2}}\sqrt{\sum_{L\in(A,C,G,T)} {({P(a)}_{i,L}{-P(b)}_{i,L})}^{2}}$$

where *m* is the motif length, *w* is the number of positions matched between two position-specific probability matrices, and ${P(a)}_{i,L}$ and ${P(b)}_{i,L}$ are probabilities of base L at position *i* in position-specific probability matrix *a* and *b*, respectively.

**Affinity Propagation Clustering Algorithm**

Affinity propagation clustering is an unsupervised clustering algorithm, which can be used when optimal number of clusters is unknown. This clustering technique works by message passing between a pair of data points. It is done through all pair-wise input data points by using four defined matrices; similarity, responsibility, availability and criterion. The messages between each pair of data points will tell how good it is suited to become an exemplar of the other. Initially, the similarity matrix is calculated from input data directly. Then, the message passing is computed based on two matrices; 1), Responsibility *R*(a, b) which tests whether ‘b’ should be exemplar for ‘a’ or not, and 2) Availability *A*(a, b) that evaluates ‘a’ should choose ‘b’ as its exemplar, as well as considering all other values for which ‘b’ should be an exemplar. Each data point will update its suitability when the responses are received from the others. Such update is continued until the convergence or the final optimal clustering are reached. For convergence of the algorithm, there are two parameters (preference and damping) need to be mentioned in training [3]: preference controls the number of exemplars, the lower the value of preference the lesser number of clusters will be generated; damping avoids numerical oscillations in the updating of responsibility and availability matrices, the higher the damping value the lesser the oscillation in the updating (e.g., a damping value equals 0.9 will converge most all of cases [4]). Since the affinity propagation clustering is most suitable for small to medium sized datasets (e.g., < a million data points) with unknown clustering numbers, it is well suited for clustering of PWMs in abc4pwm package. More information of these parameters please refer to [3] and [4].

**Pseudocode for Affinity Propagation Clustering Algorithm:**

**INPUT**: similarity matrix *S*, a set of pairwise similarities, *{S(i, k)}_(i,k)∈{1,...,N}_^2^ _,i≠ k_*

where *S(i, k)* shows how similar is point *i*  to point *k*..

**INITIALIZATION:** availability matrix *A* set to zero, for all *i, k : A(i, k)=0*.

**LOOP** in responsibility matrix *R* and availability matrix *A* until convergence (or not change) with following formula:

$R(i, k)\leftarrow S(i, k) - max \{A(i, k’) +S(i, k’)\}$ (2)

$$k’ such that k’ \neq k$$

Off-diagonal:

$A(i, k)\leftarrow\min\{0, R\left( k,k \right)+\Sigma\max\left\{ 0, R\left( i^{'},k \right) \right\}\}$ (3)

Diagonal:

$A(k, k) \leftarrow\Sigma max \{0, R(i’, k)\}$ (4)

$$i’ such that i’ \neq k$$

**OUTPUT**: Criterion matrix *C = (C_i_, . . ., C_n_)*, where

$C_{i}\leftarrow argmax[R\left( i, k \right)+ A\left( i, k \right)]$ (5)

and *C_i_* is the cluster centroid (exemplar) to which all elements *i* are assigned. For example, if there is a cluster and its exemplar is k *(C_i_)*, then all elements inside it are represented *i_._*

**Description of ideas behind equations 2, 3, and 4:**

In Sfigure3, we illustrate the relationship between elements and message passing when updating responsibility matrix *R* and availability matrix *A* in each iteration of pseudocode for affinity propagation clustering. More information of affinity propagation clustering please refer to previous paper[5].

**A step-by-step example for Affinity Propagation Clustering Algorithm.**

Here, we present an example of four matrices used in affinity propagation clustering (APC), which shows step by step calculation of the matrices in the APC for determining clustering number. STable 4 is a random input data of 5 samples (N1, N2, N3, N4, N5) with 5 features (X1, X2, X3, X4, X5), on which Affinity Propagation Clustering will be applied. This random input data is adopted from previous work [5]. STable5 shows an example of similarity matrix, an N x N similarity matrix, N is the number of samples. Based on equation (1) we obtain: (3–4)² + (4–3)² + (3–5)² + (2–1)² + (1–1)² = 7, from which the negative of this value (-7) is used as similarity between N1 and N2. In Stable 6, a responsibility matrix, based on equation (2) of main text, we get -18-(-6) = -12 for samples between N3 (row) and N4 (column). Stable 7 is the availability matrix, self-availability (diagonal values) of N1 is the sum of the positive responsibilities of N1’s column excluding N1’s self-responsibility, which is based on equation (3) from main text (e.g., 10 + 11 + 0 + 0 = 21). For off-diagonal elements of the available matrix, equation (4) from the main text is used (e.g., N1 to N2 is -15 + 0 + 0 + 0 = -15). Stable 8 is the final criterion matrix that is based on equation (5) from the main text, where there are two clusters; N1, N2 and N3 will be in one cluster while N4 and N5 will be in the second cluster.

Supplementary Tables

**STable 1: Predicted PWMs from yeast cell cycle genes that match to yeast TFs at alpha7 time point.**

PWMs of yeast cell cycle related TFs that were reported as a match for PEBMs for alpha7. Respective similarity scores for the match are also reported. As BayesPI2 can search on both strands, search for PEBMs was conducted on both strands.

| Strand | Transcription Factor | Similarity score |
| --- | --- | --- |
| strand0 | SGD-SWI4_2_psam.mlp | 0.9060529338366318 |
|  | SGD-MCM1_2_psam.mlp | 0.870009356735971 |
|  | SGD-MCM1_1_psam.mlp | 0.8356681347367713 |
|  | SGD-MCM1_1_psam.mlp | 0.8291754021357449 |
|  | SGD-NDD1_psam.mlp | 0.8198502623966897 |
|  | SGD-ACE2_2_psam.mlp | 0.8144889867221661 |
|  | SGD-ACE2_1_psam.mlp | 0.8060394187105663 |
|  | SGD-SWI5_psam.mlp | 0.8031983789542192 |
|  |  |  |
|  |  |  |
| strand1 | SGD-FKH1_1_psam.mlp | 0.8594126427727671 |
|  | SGD-ACE2_1_psam.mlp | 0.8187053238940833 |
|  | SGD-MBP1_psam.mlp | 0.8168178596749999 |
|  | SGD-MBP1_psam.mlp | 0.8157673453883034 |
|  | SGD-MCM1_2_psam.mlp | 0.809403164682961 |
|  | SGD-SWI4_2_psam.mlp | 0.8019145675961494 |
|  | SGD-NDD1_psam.mlp | 0.801830909918464 |

**STable 2: PWMs of yeast TFs matched to PEBM for alpha42, using Abc4pwm.**

PWMs of yeast cell cycle related TFs that were reported as a match for PEBMs for alpha42. Respective similarity scores for the match are also reported. As BayesPI2 can search on both strands, search for PEBMs was conducted on both strands.

| Strand | Transcription Factor | Similarity score |
| --- | --- | --- |
| strand0 | SGD-SWI4_2_psam.mlp | 0.8447413720040062 |
|  | SGD-NDD1_psam.mlp | 0.8032043615937307 |
| strand1 | SGD-MCM1_2_psam.mlp | 0.823783343380557 |
|  | SGD-MBP1_psam.mlp | 0.8197588171423683 |
|  | SGD-NDD1_psam.mlp | 0.8161802898577416 |
|  | SGD-NDD1_psam.mlp | 0.8136236250049206 |
|  | SGD-NDD1_psam.mlp | 0.8029278239421616 |

**STable 3: PWMs of yeast TFs matched to PEBM for alpha49, using Abc4pwm.**

PWMs of yeast cell cycle related TFs that were reported as a match for PEBMs for alpha49.Respective similarity scores for the match are also reported. As BayesPI2 can search on both strands, search for PEBMs was conducted on both strands.

| Strand | Transcription Factor | Similarity score |
| --- | --- | --- |
| strand0 | SGD-NDD1_psam.mlp | 0.8135017386197485 |
|  | SGD-NDD1_psam.mlp | 0.8014439744793012 |
|  | SGD-NDD1_psam.mlp | 0.8004425424217013 |
| strand1 | SGD-ACE2_2_psam.mlp | 0.8009545198749632 |
|  | SGD-NDD1_psam.mlp | 0.8005739647495393 |
|  | SGD-NDD1_psam.mlp | 0.800251548755549 |

**STable 4: Input data of 5 samples and 5 features for Affinity Propagation Clustering.**

Following is a table of input data for affinity propagation clustering, where N1 to N5 and X1 to X5 are 5 random data samples and 5 features, respectively.

| Samples | X1 | X2 | X3 | X4 | X5 |
| --- | --- | --- | --- | --- | --- |
| n1 | 3 | 4 | 3 | 2 | 1 |
| n2 | 4 | 3 | 5 | 1 | 1 |
| n3 | 3 | 5 | 3 | 3 | 3 |
| n4 | 2 | 1 | 3 | 3 | 2 |
| n5 | 1 | 1 | 3 | 2 | 3 |

**STable 5: Similarity matrix *S* inferred from the input data.**

Following table shows the values of similarities between all input samples (N x N), negative sum of squares of differences between the features, based on equation (1) in the main text.

| Samples | N1 | N2 | N3 | n4 | N5 |
| --- | --- | --- | --- | --- | --- |
| n1 | -22 | -7 | -6 | -12 | -17 |
| n2 | -7 | -22 | -17 | -17 | -22 |
| n3 | -6 | -17 | -22 | -18 | -21 |
| n4 | -12 | -17 | -18 | -22 | -3 |
| n5 | -17 | -22 | -21 | -3 | -22 |

**STable 6: Responsibility matrix *R* inferred from data similarities.**

Following is the table that shows responsibility values for input data similarities by using equation (2) of the main text.

| Samples | N1 | N2 | N3 | n4 | N5 |
| --- | --- | --- | --- | --- | --- |
| n1 | -16 | -1 | 1 | -6 | -11 |
| n2 | 10 | -15 | -10 | -10 | -15 |
| n3 | 11 | -11 | -16 | -12 | -15 |
| n4 | -9 | -14 | -15 | -19 | 9 |
| n5 | -14 | -19 | -18 | 14 | -19 |

**STable 7: Availability Matrix *A* in Affinity Propagation Clustering Algorithm.**

Following table shows example of availability matrix for the data given in STable 4. Here, the calculation of diagonal and off-diagonal values are based on equations (3) and (4) from the main text.

| Samples | N1 | N2 | N3 | n4 | N5 |
| --- | --- | --- | --- | --- | --- |
| n1 | 21 | -15 | -16 | -5 | -10 |
| n2 | -5 | 0 | -15 | -5 | -10 |
| n3 | -6 | -15 | 1 | -5 | -10 |
| n4 | 0 | -15 | -15 | 14 | -19 |
| n5 | 0 | -15 | -15 | -19 | 9 |

## **STable 8: Inferred Criterion Matrix *C* in Affinity Propagation Clustering Algorithm.**

## Following table shows an example of criterion matrix calculated based on equation (5) in main text, which all be used to classify samples to different clusters.

| Samples | N1 | N2 | N3 | n4 | N5 |
| --- | --- | --- | --- | --- | --- |
| n1 | 5 | -16 | -15 | -11 | -21 |
| n2 | 5 | -15 | -25 | -15 | -25 |
| n3 | 5 | -26 | -15 | -17 | -25 |
| n4 | -9 | -29 | -30 | -5 | -10 |
| n5 | -14 | -34 | -33 | -5 | -10 |

.

**Supplementary Figures**

**SFigure 1: A screen shot of uniform human TF DBD Database generated by abc4pwm.**

This figure shows the head rows of a newly created uniform database for human TF DBD families by using abc4pwm. First column is TF name, second column shows the source where it is taken from (e.g., HTF represents human Transcription Factor database, EW means Edger Wingender TF Classification database, JP represents JASPAR database). The third column ‘DBD Short name’ refers to name used in the uniform TF DBD database, while the last column ‘DBD full name’ refers to complete name of DBD family.


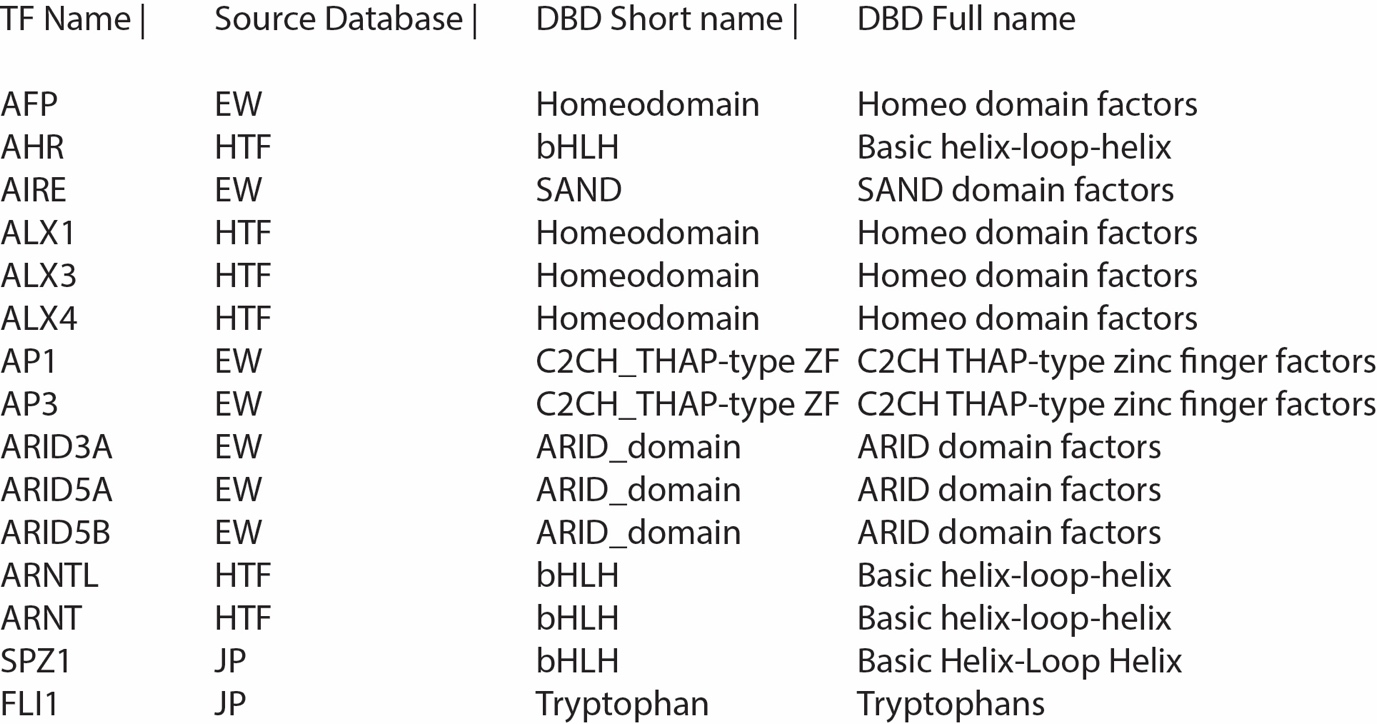


SFigure 2: Top ranked PWMs predicted in yeast cell cycle genes.

1. Table on top shows similarity score for the predicted top three PWMs for Alpha7 arrest point. Predicted PWM L12_6 and matched yeast TF SWI4 are shown in middle and bottom logo plot, respectively.
2. Table on top shows similarity score for the predicted top three motifs for Alpha49 arrest point. Predicted PWM L8_2 and matched yeast TF NDD1 are shown in middle and bottom logo plot, respectively.

**SFigure 3: A simple illustration of ideas behind equations 2, 3, and 4 in the pseudocode.**

Left side panel shows that data point *k’* and all other points send message to data point *i* stating that how well-suited (responsible) element *k’* is to be an exemplar for the element *i* (equation 2 for responsibility matrix *R*).

On the right panel, an exemplar *k* communicates with datapoint *i* and to assess how much is the data point *k* available as an exemplar for the data point *i* (equations 3 and 4 for availability matrix *A*).

_
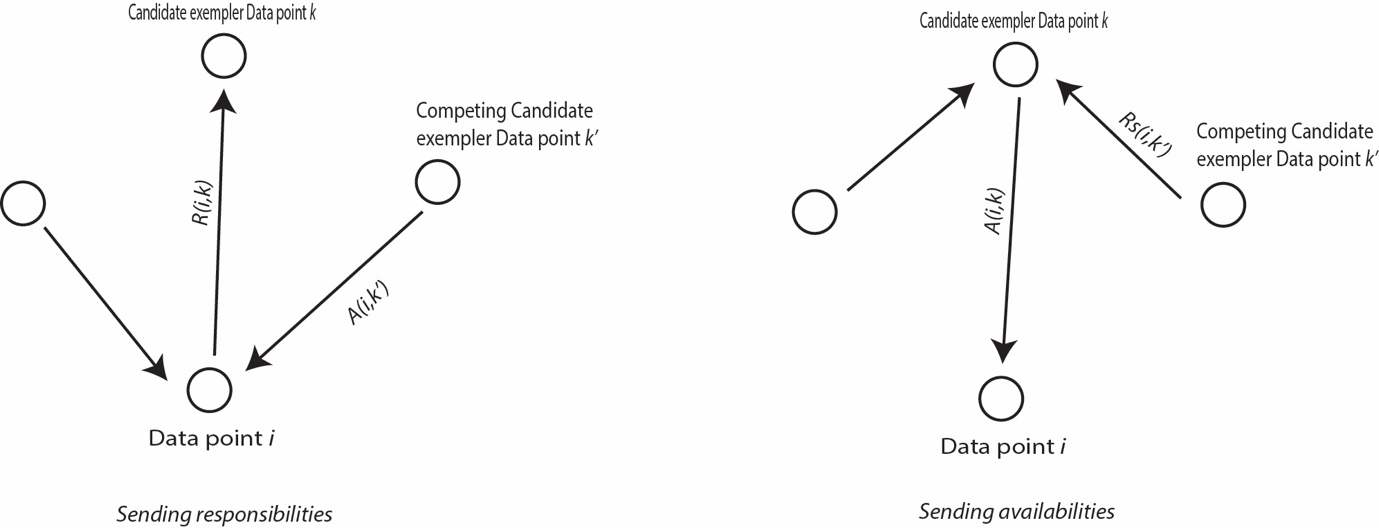
_

**Supplementary References**

1. Tsai, H.K., et al., *Method for identifying transcription factor binding sites in yeast.* Bioinformatics, 2006. **22**(14): p. 1675-81.

2. Wang, J., et al., *Comprehensive genome-wide transcription factor analysis reveals that a combination of high affinity and low affinity DNA binding is needed for human gene regulation.* BMC genomics, 2015. **16**(7): p. 1-15.

3. Frey, B.J. and D. Dueck, *Clustering by passing messages between data points.* science, 2007. **315**(5814): p. 972-976.

4. Dueck, D., *Affinity propagation: clustering data by passing messages*. 2009: Citeseer.

5. Thavikulwat, P. *Affinity propagation: A clustering algorithm for computer-assisted business simulations and experiential exercises*. in *Developments in Business Simulation and Experiential Learning: Proceedings of the Annual ABSEL conference*. 2008.
